# DLBWE-Cys: a deep-learning-based tool for identifying cysteine S-carboxyethylation sites using binary-weight encoding

**DOI:** 10.3389/fgene.2024.1464976

**Published:** 2025-01-08

**Authors:** Zhengtao Luo, Qingyong Wang, Yingchun Xia, Xiaolei Zhu, Shuai Yang, Zhaochun Xu, Lichuan Gu

**Affiliations:** ^1^ School of Information and Artificial Intelligence, Anhui Agricultural University, Hefei, Anhui, China; ^2^ Anhui Province Key Laboratory of Smart Agricultural Technology and Equipment, Hefei, Anhui, China; ^3^ Anhui Provincial Engineering Research Center for Agricultural Information Perception and Intelligent Computing, Anhui Agricultural University, Hefei, Anhui, China; ^4^ Computer Department, Jingdezhen Ceramic University, Jingdezhen, China; ^5^ School for Interdisciplinary Medicine and Engineering, Harbin Medical University, Harbin, China

**Keywords:** S-carboxyethylation, post-translational modification, bahdanau attention mechanism, binary-weight encoding, deep learning

## Abstract

Cysteine S-carboxyethylation, a novel post-translational modification (PTM), plays a critical role in the pathogenesis of autoimmune diseases, particularly ankylosing spondylitis. Accurate identification of S-carboxyethylation modification sites is essential for elucidating their functional mechanisms. Unfortunately, there are currently no computational tools that can accurately predict these sites, posing a significant challenge to this area of research. In this study, we developed a new deep learning model, DLBWE-Cys, which integrates CNN, BiLSTM, Bahdanau attention mechanisms, and a fully connected neural network (FNN), using Binary-Weight encoding specifically designed for the accurate identification of cysteine S-carboxyethylation sites. Our experimental results show that our model architecture outperforms other machine learning and deep learning models in 5-fold cross-validation and independent testing. Feature comparison experiments confirmed the superiority of our proposed Binary-Weight encoding method over other encoding techniques. t-SNE visualization further validated the model’s effective classification capabilities. Additionally, we confirmed the similarity between the distribution of positional weights in our Binary-Weight encoding and the allocation of weights in attentional mechanisms. Further experiments proved the effectiveness of our Binary-Weight encoding approach. Thus, this model paves the way for predicting cysteine S-carboxyethylation modification sites in protein sequences. The source code of DLBWE-Cys and experiments data are available at: https://github.com/ztLuo-bioinfo/DLBWE-Cys.

## 1 Introduction

Cysteine S-carboxyethylation (Zhai et al., 2023) is a unique post-translational modification in which the thiol group (-SH) of cysteine residues is modified by the addition of a carboxyethyl group (-CH2-COOH). This modification is triggered by the metabolic product 3-HPA and represents a novel protein modification mechanism. Researchers such as Zhai et al. (2023) have discovered that carboxyethylated ITGA2B can induce specific autoimmune responses by producing modified neoantigens. They also found carboxylated ITGA2B in peripheral blood mononuclear cells (PBMCs) from patients with rheumatoid arthritis (RA) and systemic lupus erythematosus (SLE). Therefore, investigating the role of cysteine S-carboxyethylation in the aetiology of these diseases, or whether it is merely a concomitant phenomenon, is crucial to understanding and treating these diseases.

Although mass spectrometry and modification-specific antibodies can identify cysteine S-carboxyethylation sites (Bern et al., 2012; Na and Paek, 2015; Yates, 2015), these methods are expensive and time consuming. There is an urgent need to develop computational methods to predict potential S-carboxyethylation sites in proteins. The advancement of machine learning and deep learning technologies has not only made significant strides in disease prediction and diagnosis (Zhang et al., 2023), personalized treatment planning (Chen et al., 2023), medical image analysis (Shen et al., 2017), and drug discovery (Drews, 2000), but also shown tremendous potential in predicting post-translational modification sites. This provides a solid theoretical foundation for constructing efficient models (Dou et al., 2021; Ertelt et al., 2024; Meng et al., 2022). Meanwhile, the use of high-throughput sequencing technologies and specific chemical probes has accumulated a large amount of relevant data, providing a data foundation for building predictive models (House et al., 2017; Rodriguez and Miller, 2014; Vanella et al., 2022). Unfortunately, as the modification of cysteine by S-carboxyethylation has only recently been discovered, there are currently no dedicated models for predicting these sites, which significantly increases the difficulty of the research.

In recent years, significant progress has been made in the field of protein modification site prediction, particularly in the prediction of S-palmitoylation, S-sulfenylation and S-nitrosylation sites. For example, GPS-Palm (Ning et al., 2021) combines CNNs with a novel graph representation system to predict S-palmitoylation sites, fastSulf-DNN (Do et al., 2021) uses word embeddings and deep neural networks to predict S-sulfenylation sites, and Mul-SNO (Zhao et al., 2022) combines BiLSTM and BERT technologies to accurately predict S-nitrosylation sites. These studies have significantly improved the accuracy and efficiency of predicting functional sites in proteins, and also provide a good reference for developing S-carboxyethylation site prediction models.

This study proposes an innovative deep learning model, DLBWE-Cys, which is the first model capable of effectively identifying cysteine S-carboxyethylation modification sites in protein sequences. The model employs a binary encoding method combined with positional weights as a feature representation and uses convolutional neural networks (CNNs), bidirectional long short-term memory networks (BiLSTMs), Bahdanau attention mechanisms, and fully connected networks for final prediction. Through this approach, the model effectively captures local information and enhances the recognition of key features to achieve accurate predictions. In the experimental part, our model, DLBWE-Cys, was compared with various machine learning and deep learning models in 5-fold cross-validation and independent test dataset. At the same time, we tested the impact of different feature extraction methods on model performance and visualised the best results using t-SNE dimensionality reduction techniques. In addition, we investigated the relationship between the weights assigned by the Bahdanau attention mechanism and the positional weights in our proposed Binary-Weight encoding. Finally, we compared the impact of binary encoding with and without positional weights on model performance.

## 2 Methods

### 2.1 Benchmark datasets

In the seminal study by Zhai et al. (2023), cysteine S-carboxyethylation was first identified as a novel post-translational modification (PTM) associated with autoimmune arthritis. To further investigate this unique protein modification, we carefully selected 960 sequences containing cysteine S-carboxyethylation as our positive samples for the study. To make our dataset more comprehensive, we also downloaded additional amino acid sequences from the NCBI database (https://www.ncbi.nlm.nih.gov/) to serve as negative samples, centered around cysteine (C). Both the positive and negative sample sequences were standardised to a length of 41 amino acids to ensure uniformity in our dataset. To increase the accuracy of our analysis and reduce data redundancy, we used the CD-HIT-SET (Li and Godzik, 2006) tool to eliminate 45% of duplicate sequences. In addition, to prevent the model from being overly biased towards a particular class, we constructed a balanced training set of 1,204 samples, with an equal number of positive and negative samples. During the fivefold cross-validation, we ensured that there were no overlapping protein types between the validation sets, thereby increasing the model’s ability to generalise. In addition, to better reflect real-world application scenarios, we created an independent test set consisting of a small number of positive samples and a larger number of negative samples, totalling 913 sequences with a ratio of 1:10. This independent test set was used to evaluate the generalisation performance of the model. Further details can be found in Table 1.

**TABLE 1:** Details of the datasets.

Datasets	Positive:Negative	Total
Training	1:1	1,204
Independent	1:10	913

### 2.2 Binary-weight Encoding (BWE)

In this study, we explore the digital representation of protein sequence information, specifically through a binary encoding approach. It has also already been used in different types of PTM site prediction, including malonylation (Chung et al., 2020), acetylation (Basith et al., 2022), succinylation (Ning et al., 2018) and formylation (Sohrawordi and Hossain, 2022), because of its simplicity and effectiveness. Our focus is on protein sequences containing the 20 amino acids ‘ACDEFGHIKLMNPQRSTVWY’, assigning each amino acid a unique 20-dimensional feature vector. For instance, alanine (A) is encoded as ‘10000000000000000000′, cysteine (C) as ‘01000000000000000000′, and tyrosine (Y) as ‘00000000000000000001'.

To further enrich the expression of sequence information, we introduced the concept of positional weighting, inspired by the phenomenon of signal towers transmitting signals in a progressively diminishing manner, akin to 1D Gaussian Noising. Imagining the central position of the protein sequence as the signal source, we observed that the signal strength exponentially decays with increasing distance from the source. Based on this observation, we use the following formula to calculate the signal strength at each position in the sequence, thereby determining the weight of the amino acid at that position $W_{i}$ :

$$W_{i}=p\times e^{-\alpha \left|d_{i}\right|}$$

where p represents the initial strength of the signal source, α is the decay coefficient, $d_{i}$ is the distance of the i th position from the central point. Subsequently, we multiply the binary encoding feature $B_{i}$ of the amino acid at each position by its weight $W_{i}$ to obtain the weighted binary encoding feature $X_{i}$ :

$$X_{i}=B_{i}\times W_{i}$$

Through this method, we not only retain the original information of the protein sequence but also introduce the relative importance of each amino acid position through positional weighting, effectively enhancing the expression of sequence information.

### 2.3 Framework overview

**FIGURE 1:**
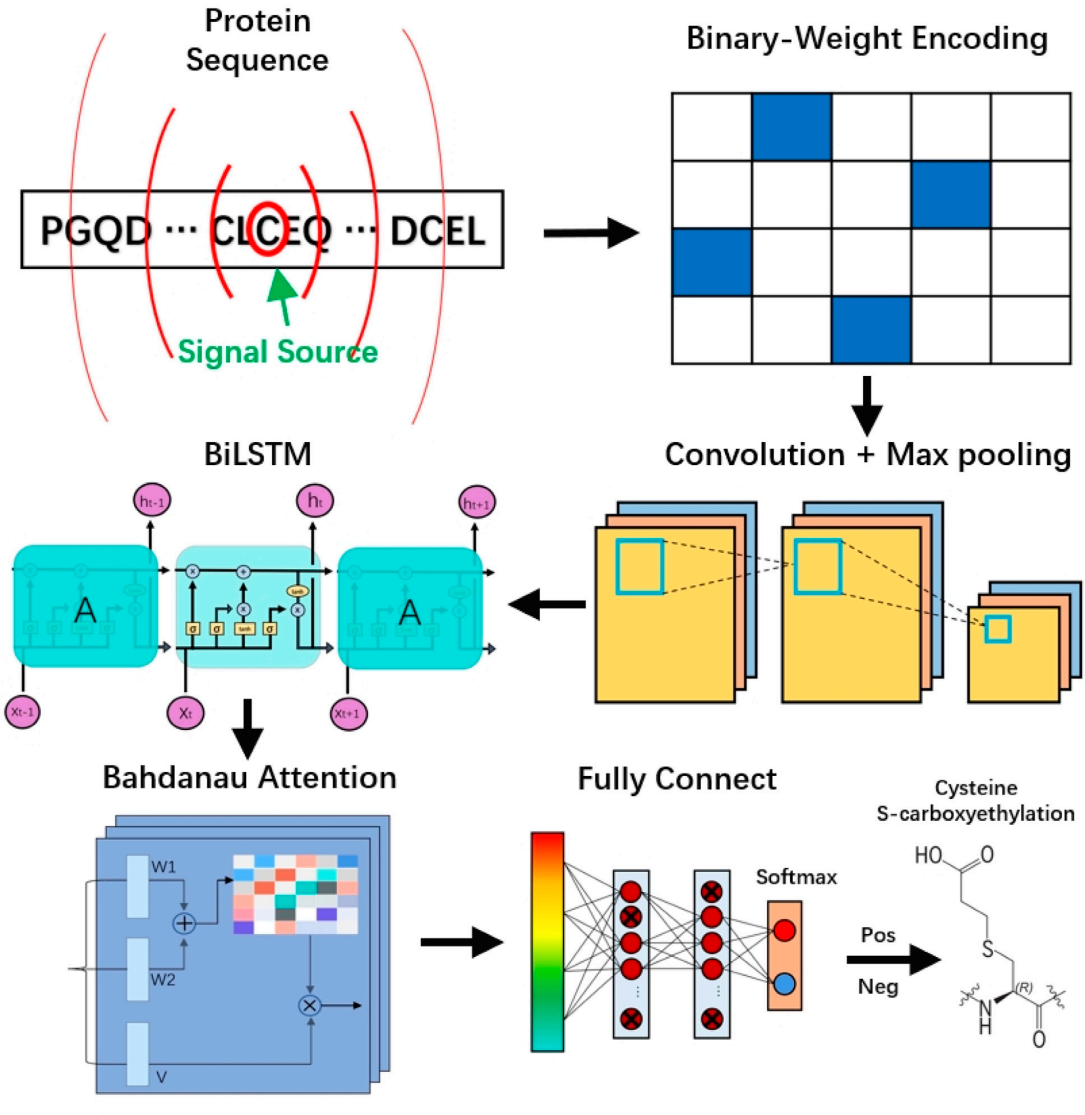
The workflow and architecture of DLBWE-Cys.

Here, we propose a novel predictive framework, DLBWE-Cys, based on CNN, BiLSTM, attention mechanisms, and FNN, which fuse protein sequence information to predict cysteine S-carboxyethylation sites. The model architecture consists of five main components, as shown in Figure 1. First, in the feature extraction module, we use the BWE method to convert protein sequences into a digital format recognizable by computers. Next, in the convolutional neural network (CNN) module, the features are fed into a network with two convolutional layers to extract local features of the protein sequence, followed by a max-pooling layer to reduce the dimensionality and computational complexity of the features.

Then, in the bidirectional long short-term memory (BiLSTM) module, unlike traditional unidirectional LSTM, BiLSTM can capture the forward and backward contextual relationships, thus providing a more comprehensive understanding of the relationships between features. In addition, in the Bahdanau attention mechanism module, we build an attention layer with three functions (W1, W2, and V) to determine the relative importance of different features. Finally, in the prediction module, the system consists of two fully connected layers and a softmax activation function. Each fully connected layer uses dropout techniques to mitigate overfitting problems, and the softmax function is used to predict whether the target amino acid C undergoes S-carboxyethylation modification.

#### 2.3.1 CNN module

We have configured the convolutional neural network module with two one-dimensional convolutional layers and a max-pooling layer. One-dimensional convolution works by applying filters to local regions of the sequence, capturing local dependencies within the sequence and thereby improving the performance of the network. Additionally, because one-dimensional convolution operates along only one dimension, it uses fewer parameters than two-dimensional convolution, making model training faster. The formula for the convolutional layer in the architecture is:

$${Conv1D\left(S\right)}_{kp}=\sum_{j=0}^{J-1}\sum_{l=0}^{L-1}W_{lj}^{p}S_{k+l,j}$$

where S represents the segment of the input, p represents the index of the kernel, and k represents the position index of the output. Further, $W^{p}$ denotes the filter with L×J weight matrix, where L denotes filter size while J denotes input channels.

In addition, to further extract key features and reduce feature dimensionality, we have incorporated a max-pooling layer into the network. With this design, we not only retain important feature information but also effectively reduce the complexity of the model.

#### 2.3.2 BiLSTM module

We use a bidirectional long short-term memory (BiLSTM) network layer to form the bidirectional long short-term memory module. BiLSTM is a unique type of recurrent neural network (RNN) that cleverly combines forward LSTM with backward LSTM, enabling it to consider both past and future information simultaneously (Hochreiter and Schmidhuber, 1997; Schuster and Paliwal, 1997). This network processes sequence information using the forward LSTM, while capturing backward temporal sequence information using the backward LSTM for a more complete understanding of context. LSTMs consist of three primary gates: the input gate, the forget gate, and the output gate, which control whether information is forgotten, stored, or passed on to the next time step.

Forget Gate:

$$f_{t}=\sigma \left(W_{f}\cdot \left[h_{t-1},x_{t}\right]+b_{f}\right)$$

Where $f_{t}$ is the activation vector of the forget gate, σ represents the sigmoid function, $W_{f}$ is the weight matrix for the forget gate, $h_{t-1}$ is the hidden state from the previous time step, $x_{t}$ is the input at the current time step, and $b_{f}$ is the bias of the forget gate.

Input Gate:

$$i_{t}=\sigma \left(W_{i}\cdot \left[h_{t-1},x_{t}\right]+b_{i}\right)$$

$${\tilde{C}}_{t}=\tan h\left(W_{C}\cdot \left[h_{t-1},x_{t}\right]+b_{C}\right)$$

Where $i_{t}$ is the activation vector of the input gate, and ${\tilde{C}}_{t}$ is the candidate cell state, activated by the tanh function.

Cell State Update:

$$C_{t}=f_{t}*C_{t-1}+i_{t}*{\tilde{C}}_{t}$$

Where $C_{t}$ is the cell state at the current time step, $C_{t-1}$ is the cell state from the previous time step, * represents element-wise multiplication.

Output Gate:

$$o_{t}=\sigma \left(W_{o}\cdot \left[h_{t-1},x_{t}\right]+b_{o}\right)$$

$$h_{t}=o_{t}*\tanh\left(C_{t}\right)$$

Where $o_{t}$ is the activation vector of the output gate, and $h_{t}$ is the hidden state at the current time step.

The backward LSTM follows the same computational process as the forward LSTM, but it starts from the last element of the sequence and processes the information in reverse order, up to the first element. The BiLSTM combines the forward and backward hidden states at each time step by concatenating them (i.e., $H_{t}=\left[h_{t},h_{t}^{\prime }\right]$ ), merging past and future information. This concatenation not only provides a richer set of sequence features but also enables the model to more comprehensively understand the entire sequence context (Liu and Guo, 2019).

#### 2.3.3 Bahdanau attention mechanism module

In our study, we have implemented an enhanced version of the Bahdanau attention mechanism (Bahdanau et al., 2014), which is designed to augment the decoder’s performance through dynamic attention to the input data (Lin and Wang, 2024). This mechanism learns to allocate weights to different encoder output features given the current state of the decoder. Specifically, we first apply linear transformations to the outputs of the BiLSTM at each timestep to obtain the intermediate vectors $W_{1}$ , $W_{2}$ , and V :

$$W_{1}=w_{1}O$$

$$W_{2}=w_{2}H$$

V=vO

Where $w_{1}$ , $w_{2}$ and v represent trainable weight matrices, O denotes the sequence of outputs from the BiLSTM, and H represents the current hidden state of the decoder. Next, we compute the attention scores as follows:

$$Scores=V^{T}\tanh\left(W_{1}+W_{2}\right)$$

To scale down the differences between the weights, we use the softmax function to normalize the scores matrix, thereby obtaining the weights for each encoder output. Finally, by applying the attention scores as weights to the outputs of the BiLSTM, we obtain the final weighted feature matrix C :

$$C=softmax\left(Scores\right)\times O$$

Through this method, the Bahdanau attention mechanism not only deepens the model’s understanding of the input data but also enhances the decoder’s ability to focus on key information during the processing phase (Lee et al., 2020).

### 2.4 Performance measures

In our past experience we have used Accuracy (ACC), Matthews correlation coefficient (MCC) (Chicco et al., 2021), Sensitivity (SN) and Specificity (SE) to evaluate the performance of different models. These measures are defined as follows:

$$Sn=\frac{TP}{TP+FN}$$

$$Sp=\frac{TN}{TN+FP}$$

$$Acc=\frac{TP+TN}{TP+TN+FP+FN}$$

$$MCC=\frac{TP\times TN-FP\times FN}{\sqrt{\left(TP+FN\right)\times \left(TN+FN\right)\times \left(TP+FP\right)\times \left(TN+FP\right)}}$$

where *TP*, *FN*, *TN*, and *FP* stand for the numbers of true positives, false negatives, true negatives, and false positives, respectively. In addition, we often calculate the area under the receiver operating characteristic curve (AUROC) and the area under the precision-recall curve (AUPR) to assess predictive performance. Higher values of AUROC and AUPR indicate better predictive performance.

## 3 Results

### 3.1 Comparing different model architectures

To demonstrate the superior performance of our model architecture, we used training dataset from the ‘benchmark datasets’ described in Section 2.1 and employed the same feature representation method, Binary-Encoding, to compare against various model architectures. These comparisons included traditional machine learning models such as Random Forest (RF), Support Vector Machines (SVM), and Extreme Gradient Boosting (XGBoost), as well as deep learning models such as CNN, BiLSTM, and a combined CNN-BiLSTM model. To verify the stability of our models, we performed 10 iterations of 5-fold cross-validation and fine-tuned the parameters. Detailed configurations for each model can be found in Supplementary Note S1. Furthermore, to investigate the impact of the decay coefficient *a* on our model, we established two comparison groups: one with the decay coefficient applied to the data and another without its application. Our results show that the application of this coefficient consistently leads to superior performance compared to the group without it, as detailed in Supplementary Information S2.

Table 2 shows the aggregated results of the 5-fold cross-validation for each model after applying the decay coefficient, including the precision-recall (PR) curves and the receiver operating characteristic (ROC) curves. The results show that most models have a small standard deviation, indicating good fit and stability. The comparison shows that, on average, deep learning models outperform machine learning models on critical metrics. For example, compared to RF, the CNN model shows an average improvement of 7.54% in ACC, 15.47% in MCC, 7.14% in AUROC and 9.44% in AUPR; the BiLSTM model shows an improvement of 7.31% in ACC, 15.68% in MCC, 7.04% in AUROC and 9.98% in AUPR. Notably, compared to RF, our DLBWE-Cys model improved ACC by 9.58%, MCC by 20.18%, AUROC by 8.7% and AUPR by 10.81%.

**TABLE 2:** Comparison of performance between different model architectures using 5-fold cross-validation on training data.

Model	ACC ± SD (%)	SN ± SD (%)	SP ± SD (%)	MCC ± SD	AUROC ± SD	AUPR ± SD
SVM	65.03 ± 0.16	61.51 ± 0.22	68.54 ± 0.25	0.3012 ± 0.0033	0.7111 ± 0.0006	0.7214 ± 0.0012
RF	65.98 ± 0.3	**68.26 ± 1.27**	63.7 ± 1	0.32 ± 0.0061	0.7128 ± 0.0059	0.7163 ± 0.0093
XGBoost	66.23 ± 0.38	65.53 ± 1.14	66.93 ± 1.09	0.3247 ± 0.0075	0.7104 ± 0.0055	0.7177 ± 0.0052
BiLSTM	73.29 ± 0.46	62.76 ± 1.92	83.82 ± 1.79	0.4768 ± 0.0095	0.7832 ± 0.0082	0.8161 ± 0.0094
CNN	73.52 ± 0.85	67.01 ± 1.91	80.03 ± 1.82	0.4747 ± 0.0174	0.7842 ± 0.0079	0.8107 ± 0.0076
CNN-BiLSTM	74.26 ± 0.55	64.35 ± 1.59	84.17 ± 1.81	0.4953 ± 0.0123	0.7885 ± 0.0087	0.8226 ± 0.0046
DLBWE-Cys	**75.56 ± 0.21**	65.63 ± 1.49	**85.48 ± 1.64**	**0.5218 ± 0.0064**	**0.7998 ± 0.0089**	**0.8244 ± 0.0082**

Bold values are the best values for the column.

We also performed a series of ablation tests to confirm the superiority of our model architecture. As shown in Table 2, our proposed model with attention mechanisms significantly outperformed the CNN-BiLSTM embedding architecture without attention, highlighting the ability of the attention mechanism to recognize critical information in sequences, thereby significantly improving model performance. Furthermore, the embedded deep learning models incorporating CNN and BiLSTM outperformed standalone models in terms of SP, ACC, MCC, AUROC and AUPR. This superiority is due to the embedded models combining the strengths of CNN and BiLSTM, which capture both local and long-term dependency information. Meanwhile, our model significantly outperforms single CNN models, showing average increases of 5.45% in SP, 2.04% in ACC, 4.71% in MCC, 1.56% in AUROC, and 1.37% in AUPR.

**FIGURE 2:**
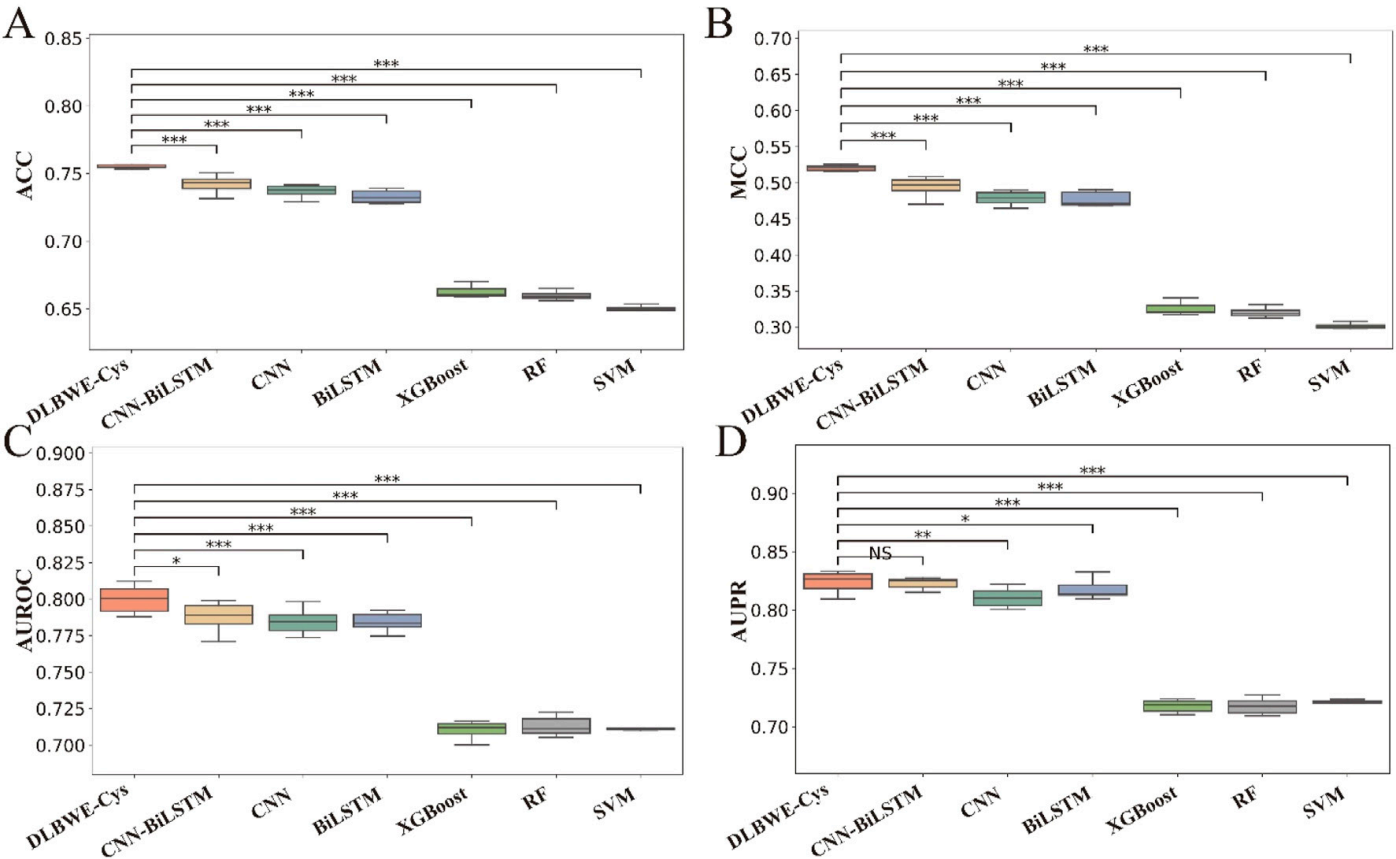
Performance analysis of different m6Am prediction models using five-fold cross-validation on training data. Subgraphs **(A–D)** represent boxplots of ACC, MCC, AUROC, and AUPR of different models, respectively. The level of significance (NS, *, **, ***) represents non-significant (*p* > 0.05), low significance (*p* < 0.05), medium significance (*p* < 0.01), and high significance (*p* < 0.001), respectively.

To visually present the differences in performance between our model and others, we used the Mann-Whitney U test to calculate the statistical significance of these differences, which are marked in Figures 2A–D using the significance levels (NS, *, **, ***). These graphs clearly show the significant differences between DLBWE-Cys and other models.

**FIGURE 3:**
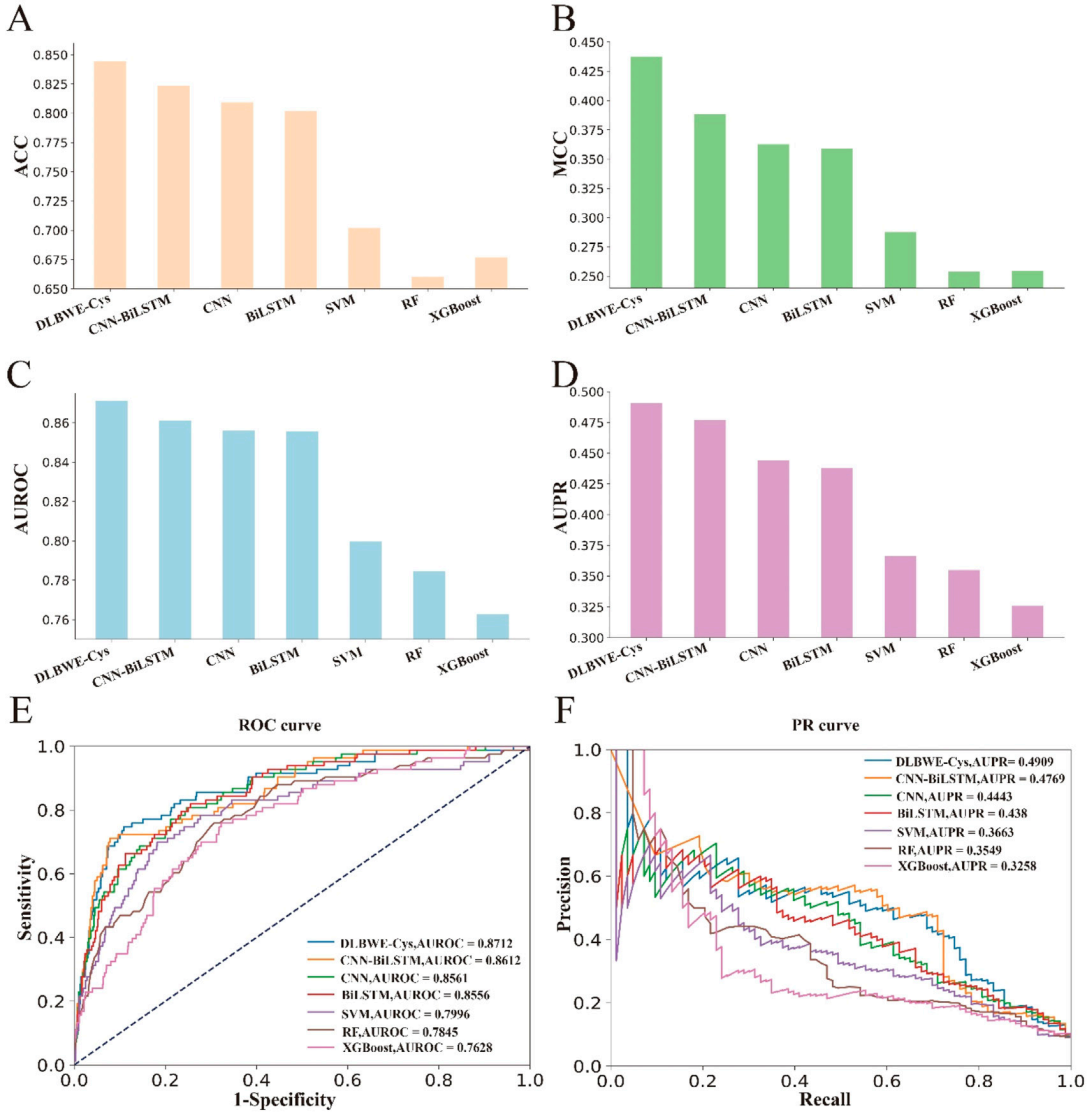
Performance comparison of DLBEW-Cys with various classical machine learning models and deep learning models on an independent test dataset. **(A–D)** represent the ACC, MCC, AUROC and AUPR values of our proposed DLBEWE-Cys in comparison with other models, including SVM, RF, XGBoost, BiLSTM, CNN and CNN-BiLSTM, respectively. **(E, F)** depict the receiver operating characteristic (ROC) curves and the precision-recall (PR) curves of the different models, respectively.

Finally, to further validate the superiority of our proposed model, we evaluated the generalization performance of DLBWE-Cys against six other models using the independent test dataset mentioned in Section 2.1. The results are presented in Supplementary Note S3. To make it easier for readers to observe the results, we have also performed a series of visualizations. Figures 3A–D shows the results for ACC, SN, AUROC, and AUPR. Additionally, Figures 3E, F provides comparisons between different methods based on PR and ROC curves. These results indicate that DLBWE-Cys generally outperforms the other models, confirming the advantages of the deep learning architecture and attention mechanism.

### 3.2 Comparing different feature representation methods

To validate the effectiveness of our BWE method in feature representation, we used the iLearnPlus (Chen et al., 2021; Chen et al., 2018; Chen et al., 2020) to select five different manual feature encoding methods for comparison. These methods include AAindex (Kawashima et al., 2008), Composition, Transition and Distribution (CTD) (Dubchak et al., 1995), Enhanced Amino Acid Composition (EAAC) (Zhou et al., 2018), Dipeptides Composition (DPC) (Bhasin and Raghava, 2004) and Tripeptides Composition (TPC) (Saravanan and Gautham, 2015), with further details available in Supplementary Note S4. We then applied each of these feature encoding methods to our model architecture for training and testing, and evaluated the differences between them using four key performance metrics: ACC, MCC, AUROC and AUPR.

**FIGURE 4:**
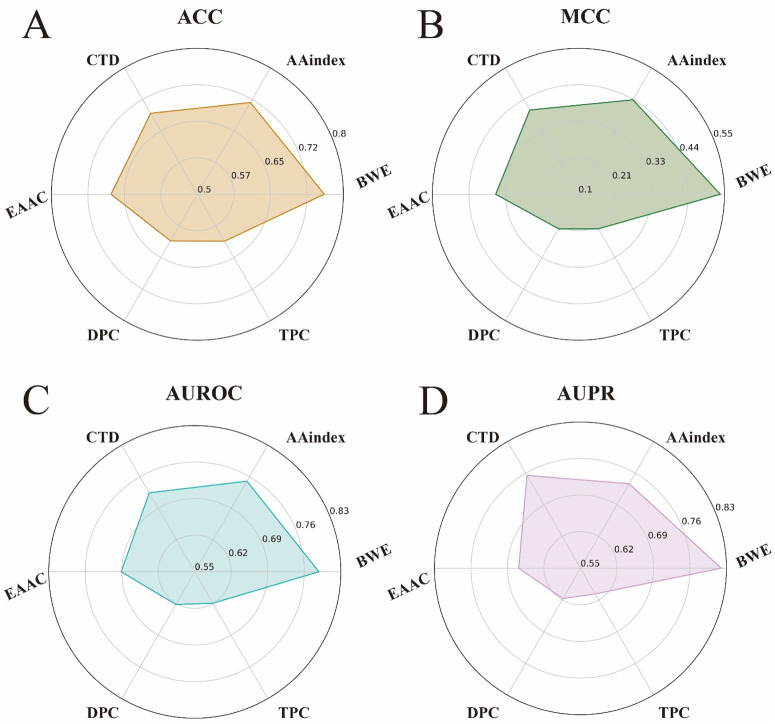
Performance comparison between the feature representation of BWE and other five handcrafted feature encoding methods. **(A–D)** respectively represent the ACC, MCC, AUROC and AUPR values of the feature representation of BWE and other handcrafted feature encoding methods, including AAindex, EAAC, CTD, DPC and TPC.

As shown in Figures 4A–D, the BWE method outperformed the other feature encoding methods on all metrics. Specifically, the BWE method showed improvements of 4.3%, 10.1%, 3.8%, and 8.34% in ACC, MCC, AUROC, and AUPR, respectively, compared to the second-best encoding method. Most notably, compared to the DPC encoding, the BWE method demonstrated increases of 15.02%, 31.45%, 16.59%, and 20.28% in ACC, MCC, AUROC, and AUPR, respectively. These results further confirm the significant advantages of our proposed BWE method.

### 3.3 Visualization of feature representation during training

**FIGURE 5:**
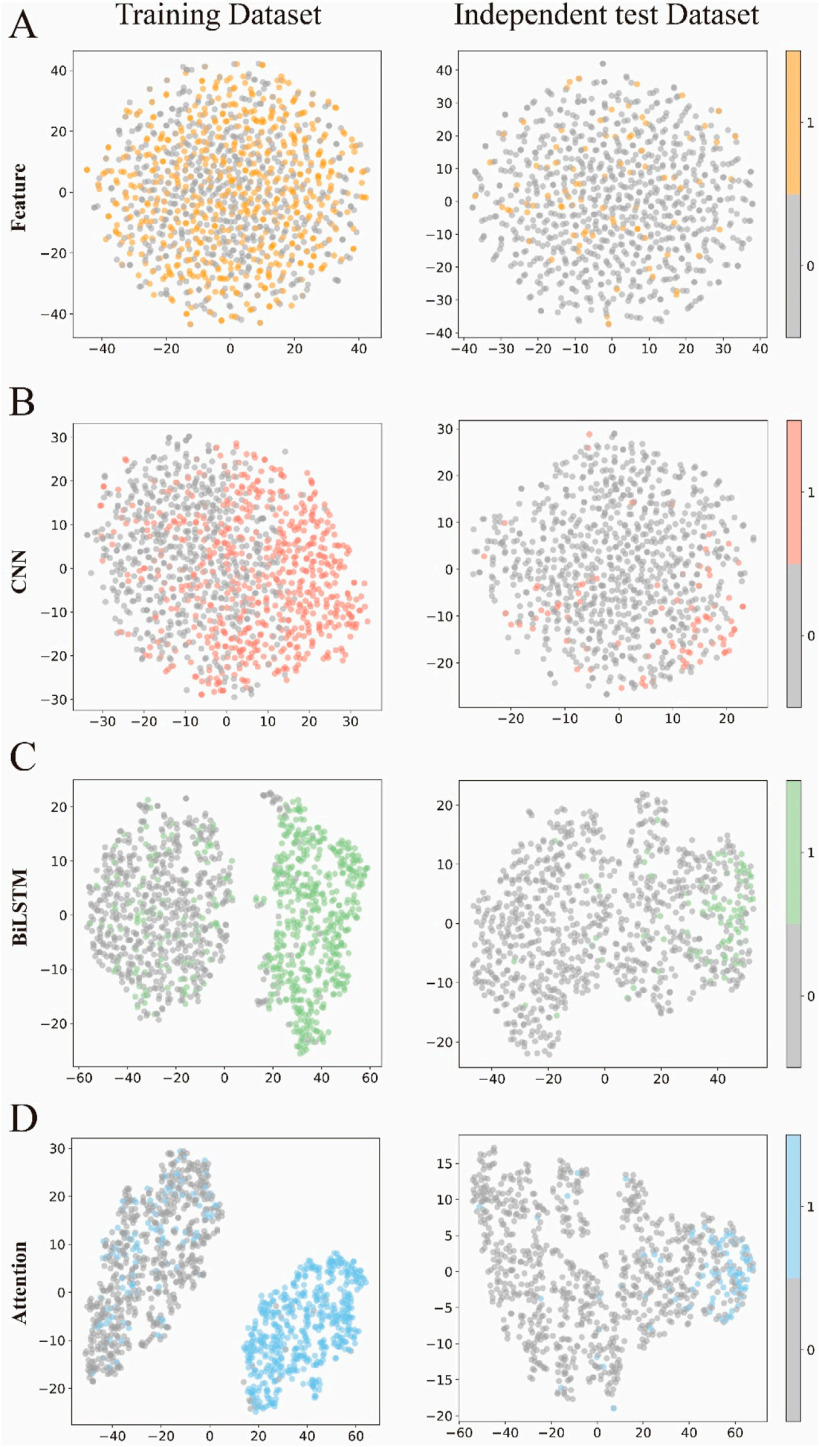
Visualization of the features of the different modules. **(A–D)** respectively represent the t-SNE visualizations of the original sequence after passing through the feature representation module, CNN module, BiLSTM module, and attention module.

To visually demonstrate how each module in DLBWE-Cys extracts different sequence features, we used the t-distributed stochastic neighbour embedding (t-SNE) (Fang et al., 2023) technique to reduce high-dimensional features to two dimensions for visualization. t-SNE is a nonlinear dimensionality reduction technique that effectively reveals the nonlinear structures of data. Figures 5A–D show the distribution of prediction probabilities in the training and independent test dataset within the feature extraction module, CNN module, BiLSTM module, and attention mechanism module, respectively.

This figure shows t-SNE plots of the data after each module - Feature Extraction, CNN, BiLSTM and Attention - arranged by rows, with each column comparing the training set with the test set. Initially, the points appear intermixed, showing limited class separation. After the CNN module, subtle clustering emerges in the training data, although this pattern is less pronounced in the test data. In contrast, the BiLSTM and attention modules produce clearer clusters during training, indicating stronger class separation. Although class separation is somewhat reduced in the test data, these modules still produce more discernible groupings than the earlier stages.

Overall, these visualisations highlight the importance of each component of the model (Pakhrin et al., 2023a; Pakhrin et al., 2023b; Palacios et al., 2024). Each successive module contributes to the extraction of more informative features, ultimately improving the model’s ability to discriminate between classes and increasing its overall recognition performance.

### 3.4 Comparison of attention weights and position weights

**FIGURE 6:**
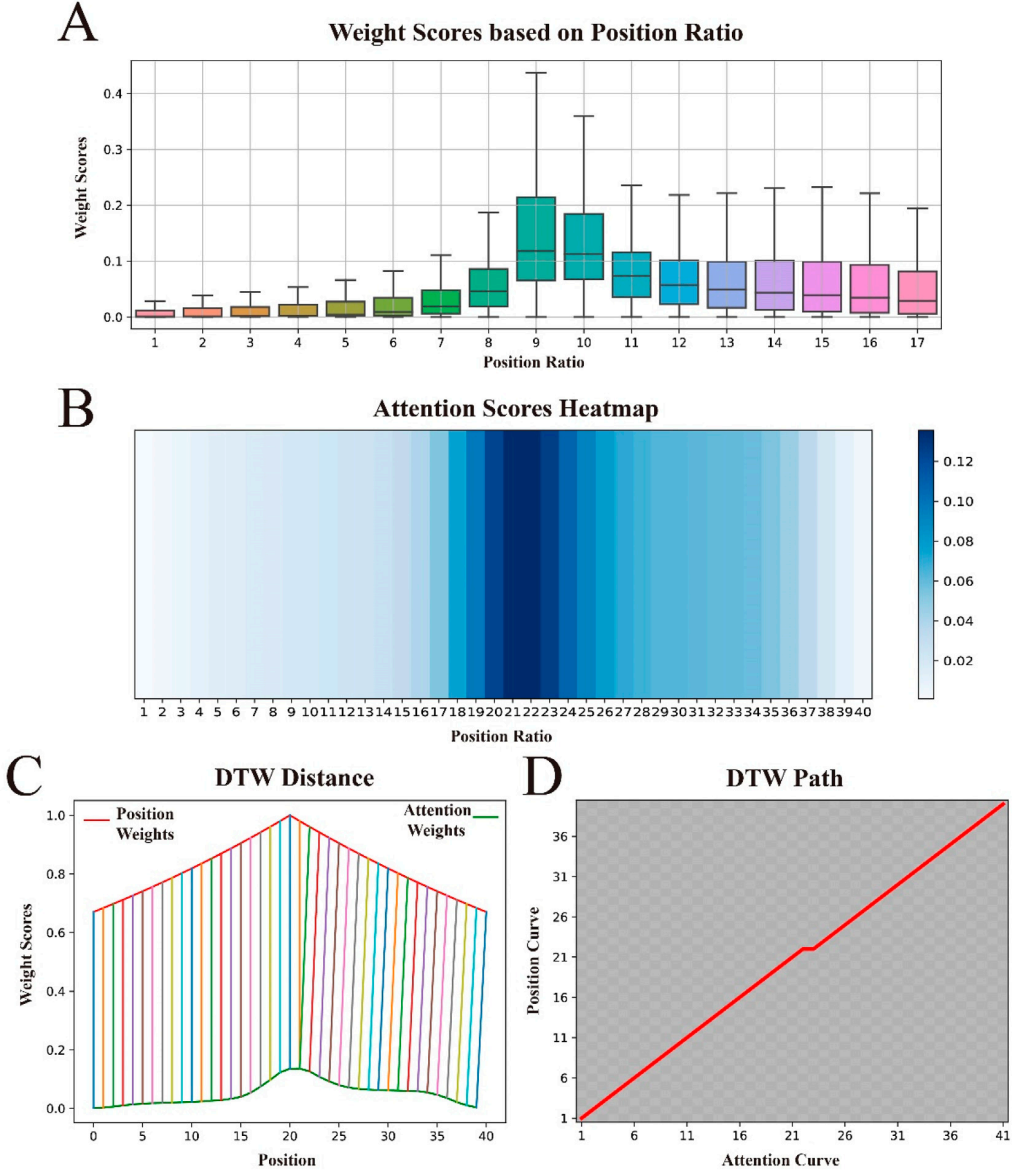
Visualization of attention weights and position weights, and their similarities. **(A)** shows the distribution of attention weights across the relative positional ratios within the sequence. **(B)** displays the distribution of attention weights “stretched” to approximate the length of the original sequence. **(C)** uses Dynamic Time Warping (DTW) to calculate the distance between the attention weight curve and the position weight curve, demonstrating their similarities. **(D)** presents the optimal alignment path between the two.

To gain a deeper understanding of how the attention mechanism assigns weights to sequence features, and to understand the contributions of different sequence positions to the model’s predictive performance, we visualized the attention weights as shown in Figure 6A. The figure shows that the attention mechanism tends to assign higher weights to the central region of the sequence, with the weights gradually decreasing as the distance from the center increases. Surprisingly, this distribution pattern is fully consistent with the concept of our proposed BEW, where weights decrease with increasing distance from the signal source.

In principle, the process of using a decay coefficient α to form a position-based weighting matrix for binary encoding is mathematically consistent with applying an attention-based weighting matrix to features after they have passed through the CNN and BiLSTM. Furthermore, since the binary-encoded features retain a similar structural organisation after processing by the CNN and BiLSTM, the comparison of the two weighting matrices remains both valid and meaningful.

To further explore the relationship between attention weights and BWE, we conducted a series of experiments. First, to better compare their similarities, we “stretched” the attention weight score matrix to match the length of the original sequence, with specific details available in Supplementary Note S5. To observe the effect of this “stretching”, we visualized it again, with darker colors in the heat map indicating higher importance scores, as shown in Figure 6B. We observed that the color gradually lightens from the center to the edges, indicating that the “stretching” operation did not change the distribution pattern of attention weights. We then used Dynamic Time Warping (DTW) (Sakoe and Chiba, 1978) to compare the similarity between the “stretched” attention weight vector and the weight vector in BWE, as shown in Figure 6C. Both curves rise at the beginning of the sequence, continue to rise towards the center and then gradually fall towards the end. Furthermore, these two curves are almost perfectly aligned at all positions and show very similar trends. Finally, to better observe the optimal alignment path of the two curves, we visualized the path matrix of both curves, as shown in Figure 6D. The path is shown as a perfect diagonal, indicating that the changes in the two curves are synchronous, i.e., the optimal alignment path is direct.

These experimental results suggest that the way we assign position weights and the way the attentional mechanism enhances important features are similar, further demonstrating the strong explanatory power of BWE in theory. In addition, it demonstrates high adaptability to complex weighting patterns in practical applications.

### 3.5 Comparison of binary encoding with and without weights

**FIGURE 7:**
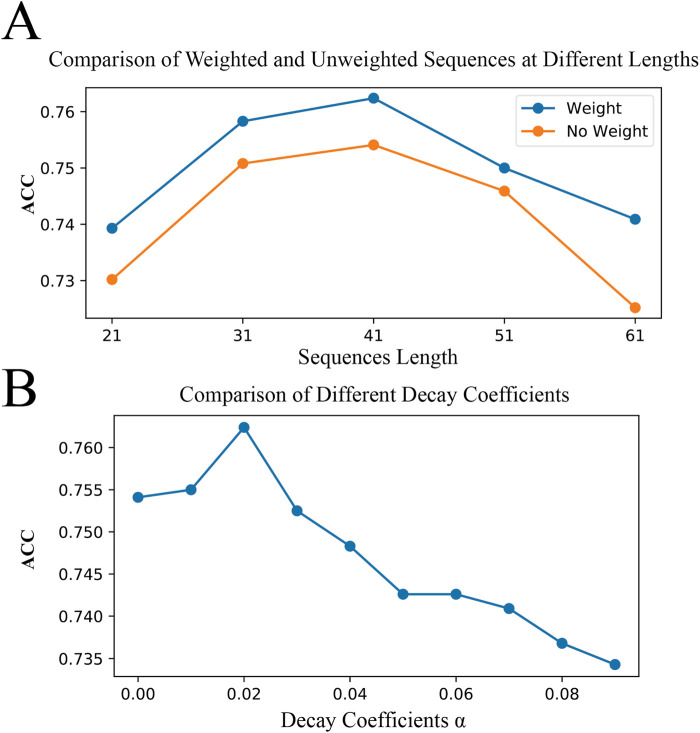
Optimal Sequence Length and Decay Coefficients. **(A)** demonstrates the impact of binary encoding with and without positional weights on model accuracy across sequence lengths of 21, 31, 41, 51, and 61 amino acids. **(B)** shows the effect of different decay coefficients on model accuracy at a sequence length of 41 amino acids.

Here, we compare the effects of binary encoding with and without position weights on model performance across various sequence lengths. To further understand the impact of our proposed BWE method on model performance, we used a controlled variable comparison method, testing binary encoding in models with and without position weights under the same model parameters and other conditions. Meanwhile, since individual test results may be random, we fixed the central position of the original sequences and constructed benchmark datasets of different lengths (21, 31, 41, 51, and 61 amino acids) to achieve an accurate evaluation of model performance, as shown in Figure 7A. We observed that for all sequence lengths tested, the models performed best at a sequence length of 41 amino acids, regardless of whether weights were applied. Furthermore, regardless of sequence length, configurations with position weights generally outperformed those without weights. In particular, at a sequence length of 41 amino acids, we found that the model performed best when the decay coefficient, α , was set to 0.02, as shown in Figure 7B. It's worth noting that the unweighted scenario corresponds to the ideal state, where the decay coefficient α is zero. This indicates that our proposed new method does indeed include this ideal case, which means that the performance of the unweighted method could not surpass that of the weighted method.

## 4 Conclusion

In this study, we proposed a deep learning architecture named DLBWE-Cys, which integrates CNN, BiLSTM, Bahdanau attention mechanism, and FNN utilizing a Binary-Weight encoding approach specifically designed for precise identification of cysteine S-carboxyethylation sites. The combination of CNN and BiLSTM effectively captures the local and long-term dependencies of sequences, while the attention mechanism enhances the detection of key features and the FNN is responsible for the final predictions.

Experimental results demonstrate a significant advantage of our model over other machine learning and deep learning models, both in 5-fold cross-validation and on the independent test dataset. Feature comparison experiments also confirmed the superiority of our proposed Binary-Weight encoding method over other encoding techniques. In addition, t-SNE visualization highlighted the model’s strong capability for accurate classification through the effective integration of different architectural components. Furthermore, we found that the distribution of attention weights was very similar to our proposed method of distributing positional weights, further validating the rationality of our approach. Finally, our experiments proved that the model performance using Binary-Weight encoding surpasses that of standard binary encoding at any sequence length, further confirming the effectiveness of our proposed improvements.

## Data Availability

The datasets presented in this study can be found in online repositories. The names of the repository/repositories and accession number(s) can be found in the article/Supplementary Material.
