# Supplementary material for: DLBWE-Cys: a deep-learning-based tool for identifying cysteine S-carboxyethylation sites using binary-weight encoding
Source: Front Genet. 2025 Jan 8;15:1464976. doi: 10.3389/fgene.2024.1464976 (PMC11751040; doi:10.3389/fgene.2024.1464976)
Supplement: Supplementary file 1 [file DataSheet1.pdf]

Supplementary Material

1 Supplementary Note S1

Supplementary Note S1 provides more details regarding actual model configurations used, such as layer sizes, depth, number of parameters, etc.

XGBoost

GradientBoostingClassifier:(loss='deviance',learning_rate=0.1,n_estimators=100, subsample=1.0,criterion='friedman_mse',min_samples_split=2,min_samples_leaf=1, min_weight_fraction_leaf=0.0,max_depth=3,min_impurity_decrease=0.0,init=None, random_state=None,max_features=None,verbose=0,max_leaf_nodes=None,warm_start=False, validation_fraction=0.1,n_iter_no_change=None,tol=0.0001,ccp_alpha=0.0)

SVM

SVC:(C=1.0,kernel='rbf',degree=3,gamma='scale',coef0=0.0,shrinking=True, probability=False,tol=0.001,cache_size=200,class_weight=None,verbose=False, max_iter=-1,decision_function_shape='ovr',break_ties=False,random_state=None)

RF

RandomForestClassifier(n_estimators=100,criterion='gini',max_depth=None, min_samples_split=2,min_samples_leaf=1,min_weight_fraction_leaf=0.0, max_features='auto',max_leaf_nodes=None,min_impurity_decrease=0.0,bootstrap=True, oob_score=False,n_jobs=None,random_state=None,verbose=0,warm_start=False, class_weight=None,ccp_alpha=0.0,max_samples=None)

CNN

```
newModel1(
(block2): Sequential(
(0): Conv1d(20, 25, kernel_size=(6,), stride=(1,))
(1): Conv1d(25, 37, kernel_size=(6,), stride=(1,))
(2): MaxPool2d(kernel_size=2, stride=2, padding=0, dilation=1, ceil_mode=False))
(block3): Sequential(
(0): ReLU()
(1): Dropout(p=0.5, inplace=False)
(2): Linear(in_features=270, out_features=20, bias=True)
(3): ReLU()
(4): Linear(in_features=20, out_features=2, bias=True)
(5): Softmax(dim=1)))
```

BiLSTM

```
newModel1(
(lstm): LSTM(20, 40, batch_first=True, bidirectional=True)
(block3): Sequential(
(0): ReLU()
```

```
(1): Dropout(p=0.5, inplace=False)
(2): Linear(in_features=3280, out_features=20, bias=True)
(3): ReLU()
(4): Linear(in_features=20, out_features=2, bias=True)
(5): Softmax(dim=1)))
```

CNN-BiLSTM

```
newModel1(
(block2): Sequential(
(0): Conv1d(20, 32, kernel_size=(6,), stride=(1,))
(1): Conv1d(32, 48, kernel_size=(6,), stride=(1,))
(2): MaxPool2d(kernel_size=2, stride=2, padding=0, dilation=1, ceil_mode=False))
(lstm): LSTM(24, 36, batch_first=True, bidirectional=True)
(block3): Sequential(
(0): ReLU()
(1): Dropout(p=0.5, inplace=False)
(2): Linear(in_features=1080, out_features=20, bias=True)
(3): ReLU()
(4): Linear(in_features=20, out_features=2, bias=True)
(5): Softmax(dim=1)))
```

DLBWE-Cys

```
newModel1(
(block2): Sequential(
(0): Conv1d(20, 64, kernel_size=(4,), stride=(1,))
(1): Conv1d(64, 128, kernel_size=(4,), stride=(1,))
(2): MaxPool2d(kernel_size=2, stride=2, padding=0, dilation=1, ceil_mode=False))
(lstm): LSTM(64, 96, batch_first=True, bidirectional=True)
(Attention): BahdanauAttention(
(W1): Linear(in_features=192, out_features=32, bias=True)
(W2): Linear(in_features=192, out_features=32, bias=True)
(V): Linear(in_features=32, out_features=1, bias=True))
(block3): Sequential(
(0): ReLU()
(1): Dropout(p=0.5, inplace=False)
(2): Linear(in_features=192, out_features=20, bias=True)
(3): ReLU()
(4): Linear(in_features=20, out_features=2, bias=True)
(5): Softmax(dim=1)))
```

2 Supplementary Note S2

We constructed two comparative groups: one group applied the decay coefficient to the data, while the other group did not. Supplementary **Table S1** shows the results of 5-fold cross-validation for each model without the decay coefficient, while Supplementary **Table S2** presents the performance improvement of the models with the decay coefficient, calculated using the following formula:

$$Improvement\ Scores = \frac{Original\ value - Old\ value}{Old\ value} \times 100\% \quad (1)$$

In addition, we determined the optimal decay coefficient for each model. Notably, the model with the best performance is our proposed DLBWE-Cys, which combines the Bahdanau attention mechanism, CNN, and BiLSTM, and achieves the best results when the decay coefficient is applied.

Table S1. 5-fold cross-validation results for each model without the application of the decay coefficient.

model	ACC ± SD (%)	SN ± SD (%)	SP ± SD (%)	MCC ± SD	AUROC ± SD	AUPR ± SD
SVM	64.66±0.4	61.5±0.77	67.83±1.31	0.2938±0.0083	0.707±0.0051	0.7184±0.0047
RF	65.39±0.42	67.76±1.1	63.02±1.21	0.3082±0.0084	0.7086±0.0057	0.7103±0.0078
XGBoost	65.92±0.22	65.02±0.96	66.83±1	0.3186±0.0044	0.7097±0.0052	0.7173±0.005
BiLSTM	72.83±0.59	63.21±1.98	82.46±1.73	0.4656±0.0117	0.7779±0.0086	0.8102±0.0082
CNN	72.85±1.05	65±3.5	80.7±3.62	0.4639±0.0228	0.7814±0.0111	0.8083±0.0154
CNN-BiLSTM	73.7±0.58	65.81±3.2	81.6±3.83	0.4813±0.0144	0.7888±0.0092	0.8171±0.0114
CNN-BiLSTM-Attention	74.94±0.32	64.82±1.85	85.07±1.7	0.5097±0.0064	0.7847±0.0101	0.8094±0.0151

Table S2. Performance improvement of each model in 5-fold cross-validation with the application of the optimal decay coefficient.

model	ACC	SN	SP	MCC	AUROC	AUPR	Best α
SVM	0.57%	0.02%	1.05%	2.52%	0.58%	0.42%	0.02
RF	0.90%	0.74%	1.08%	3.83%	0.59%	0.84%	0.01
XGBoost	0.47%	0.78%	0.15%	1.91%	0.10%	0.06%	0.03
BiLSTM	0.63%	-0.71%	1.65%	2.41%	0.68%	0.73%	0.04
CNN	0.92%	3.09%	-0.83%	2.33%	0.36%	0.30%	0.03
CNN-BiLSTM	0.76%	-2.22%	3.15%	2.91%	-0.04%	0.67%	0.02
DLBWE-Cys	0.83%	1.25%	0.48%	2.37%	1.92%	1.85%	0.02

3 Supplementary Note S3

Supplementary note S3 provides the results of the independent test experiments, details of which are given in **Table S3**.

Table S3. Performance of different model architectures on the independent test dataset.

model	ACC (%)	SN (%)	SP (%)	MCC	AUROC	AUPR
XGBoost	67.69	75.9	66.87	0.2546	0.7628	0.3258
RF	66.05	78.31	64.82	0.2541	0.7845	0.3549
SVM	70.21	78.31	69.4	0.2877	0.7996	0.3663
BiLSTM	80.18	72.29	80.96	0.3591	0.8556	0.438
CNN	80.94	71.08	81.93	0.3627	0.8561	0.4443
CNN-BiLSTM	82.37	72.29	83.37	0.3883	0.8612	0.4769
**DLBWE-Cys**	**84.45**	**75.9**	**85.3**	**0.4377**	**0.8712**	**0.4909**

4 Supplementary Note S4

Supplementary Note S4 provides details of each handcrafted feature code, including DPC, TPC, EAAC, AAindex, and CTD.

DPC

Dipeptide Composition (DPC) analyzes the frequency of adjacent amino acid pairs in protein sequences. Since proteins contain 20 amino acids, there are 400 possible dipeptide combinations. The frequencies of these 400 dipeptides are used as the feature matrix.

TPC

Tripeptide Composition (TPC) analyzes the combinations of three consecutive amino acids (tripeptides) in protein sequences, generating features for 8,000 different tripeptide combinations. This method involves traversing the protein sequence and counting the frequency of each tripeptide to form a feature matrix.

EAAC

The Enhanced Amino Acid Composition (EAAC) method improves upon the traditional AAC approach by considering the combinations of each amino acid and its neighboring amino acids within a defined window size (a length of 5 was chosen for this experiment). This method calculates and captures the local feature frequencies in the sequence.

CTD

Composition, Transition, and Distribution (CTD) classifies the 20 amino acids into three categories based on their respective properties. **Table S4** provides detailed information about the division of amino acids. Composition (C) refers to the overall percentage composition of the 20 natural amino acids. Transition (T) characterizes the frequency percentage of changing from one natural amino acid to another. Distribution (D) refers to the positions of the first, 25%, 50%, 75%, and 100% occurrence of amino acids with certain characteristics within a protein sequence.

AAindex

The primary physicochemical properties, based on amino acid indices, were extracted from the AAindex database. In this study, for a protein fragment of 41 residues, each amino acid position is represented by 12 values related to its physicochemical and biochemical properties. These include: net charge(KLEP840101), normalized frequency of alpha-helix(MAXF760101), alpha helix propensity at position 44 in T4 lysozyme(BLAM930101), amino acid composition in intracellular proteins(CEDJ970104), amino acid composition of membrane in multi-spanning proteins(NAKH920108), volume of crystallographic water(TSAJ990101), information value for accessibility(BIOV880101), transfer energy between organic solvent and water(NOZY710101), amino acid composition of membrane proteins(NAKH900109), entropy of formation(HUTJ700103), conformational preference for all beta-strands(LIFS790101), and optimized relative partition energies(MIYS990104). **Table S5** provides detailed information.

Table S4. Division of the 20 natural amino acids according to different physicochemical properties.

Physicochemical properties	Class 1	Class 2	Class 3
Hydrophobicity	DEKNQR	AGHPSTY	CFILMVW
Normalized van der Waals volume	ACDGPST	EILNQV	FHKMRWY
Polarity	CFILMVWY	AGPST	DEHKNQR
Polarizability	ADGST	CEILNPQV	FHKMRWY
Charge	KR	DE	ACFGHIMNPQSTVWY
Secondary structures	AEHKLMQR	CFITVWY	DGNPS
Solvent accessibility	ACFGILVW	DEKNQR	HMPSTY

Table S5. Detailed physicochemical and biochemical property Indices of amino acids from the AAindex database.

AccNo	TSAJ990101	MAXF760101	NAKH920108	BLAM930101	BIOV880101	CEDJ970104	NOZY710101	KLEP840101	NAKH900109	LIFS790101	HUTJ700103	MIYS990104
A	89.3	1.43	9.36	0.96	16	7.9	0.5	0	9.25	0.92	154.33	-0.04
R	190.3	1.18	0.27	0.77	-70	4.9	0	1	3.96	0.93	341.01	0.07
N	122.4	0.64	2.31	0.39	-74	4	0	0	3.71	0.6	207.9	0.13
D	114.4	0.92	0.94	0.42	-78	5.5	0	-1	3.89	0.48	194.91	0.19
C	102.5	0.94	2.56	0.42	168	1.9	0	0	1.07	1.16	219.79	-0.38
E	138.8	1.67	0.94	0.53	-106	7.1	0	-1	4.8	0.61	223.16	0.23
Q	146.9	1.22	1.14	0.8	-73	4.4	0	0	3.17	0.95	235.51	0.14
G	63.8	0.46	6.17	0	-13	7.1	0	0	8.51	0.61	127.9	0.09
H	157.5	0.98	0.47	0.57	50	2.1	0.5	0	1.88	0.93	242.54	-0.04
I	163	1.04	13.73	0.84	151	5.2	1.8	0	6.47	1.81	233.21	-0.34
L	163.1	1.36	16.64	0.92	145	8.6	1.8	0	10.94	1.3	232.3	-0.37
K	165.1	1.27	0.58	0.73	-141	6.7	0	1	3.5	0.7	300.46	0.33
M	165.8	1.53	3.93	0.86	124	2.4	1.3	0	3.14	1.19	202.65	-0.3
F	190.8	1.19	10.99	0.59	189	3.9	2.5	0	6.36	1.25	204.74	-0.38
P	121.6	0.49	1.96	-2.5	-20	5.3	0	0	4.36	0.4	179.93	0.19
S	94.2	0.7	5.58	0.53	-70	6.6	0	0	6.26	0.82	174.06	0.12
T	119.6	0.78	4.68	0.54	-38	5.3	0.4	0	5.66	1.12	205.8	0.03
W	226.4	1.01	2.2	0.58	145	1.2	3.4	0	2.22	1.54	237.01	-0.33
Y	194.6	0.69	3.13	0.72	53	3.1	2.3	0	3.28	1.53	229.15	-0.29
V	138.2	0.98	12.43	0.63	123	6.8	1.5	0	7.55	1.81	207.6	-0.29

5 Supplementary Note S5

In **Supplementary Note S1**, concerning "**DLBWE-Cys**", we learned that each sequence corresponds to a 1x17 dimensional attention weight vector. To accurately "expand" this vector, we studied how the original feature matrix was "reduced". As shown in **Figure S1(A)**, each sequence initially represents a 41x20 dimensional feature matrix (f1). After processing through the first convolutional layer, this matrix is transformed into 37x64 dimensions (f2), and then further into 35x128 dimensions (f3) after the second convolutional layer. Finally, after processing through a max pooling layer, it becomes a 17x64 dimensional matrix (f4). It is important to note that the BiLSTM layer does not alter the length of the sequence, and hence it is omitted from this analysis.

Since the one-dimensional convolution and max pooling operations preserve the temporal structure of the data, the weight matrix allocated by the attention mechanism also retains this characteristic. Consequently, based on this feature of the weight matrix, we adopted a uniform distribution method to expand the attention weight vector, aiming to approximate the length of the original sequence. The specific steps are as follows: First, we duplicate the attention weight vector a1 to form a 1x34 dimensional vector a2, which simulates the state before the max pooling layer. Subsequently, each dimension of this vector is divided into four equal parts, creating a 4x34 dimensional vector. By accumulating the values at corresponding positions, we obtain a 1x37 dimensional weight vector a3, thus restoring the state prior to the second convolutional layer. The restoration to the state before the first convolutional layer is similar to the previous step and will not be elaborated here; ultimately, we obtain a 1x40 dimensional weight vector a4, as detailed in **Figure S1(B)**. Through these steps, we successfully generated an attention weight vector that closely approximates the length of the input sequence.

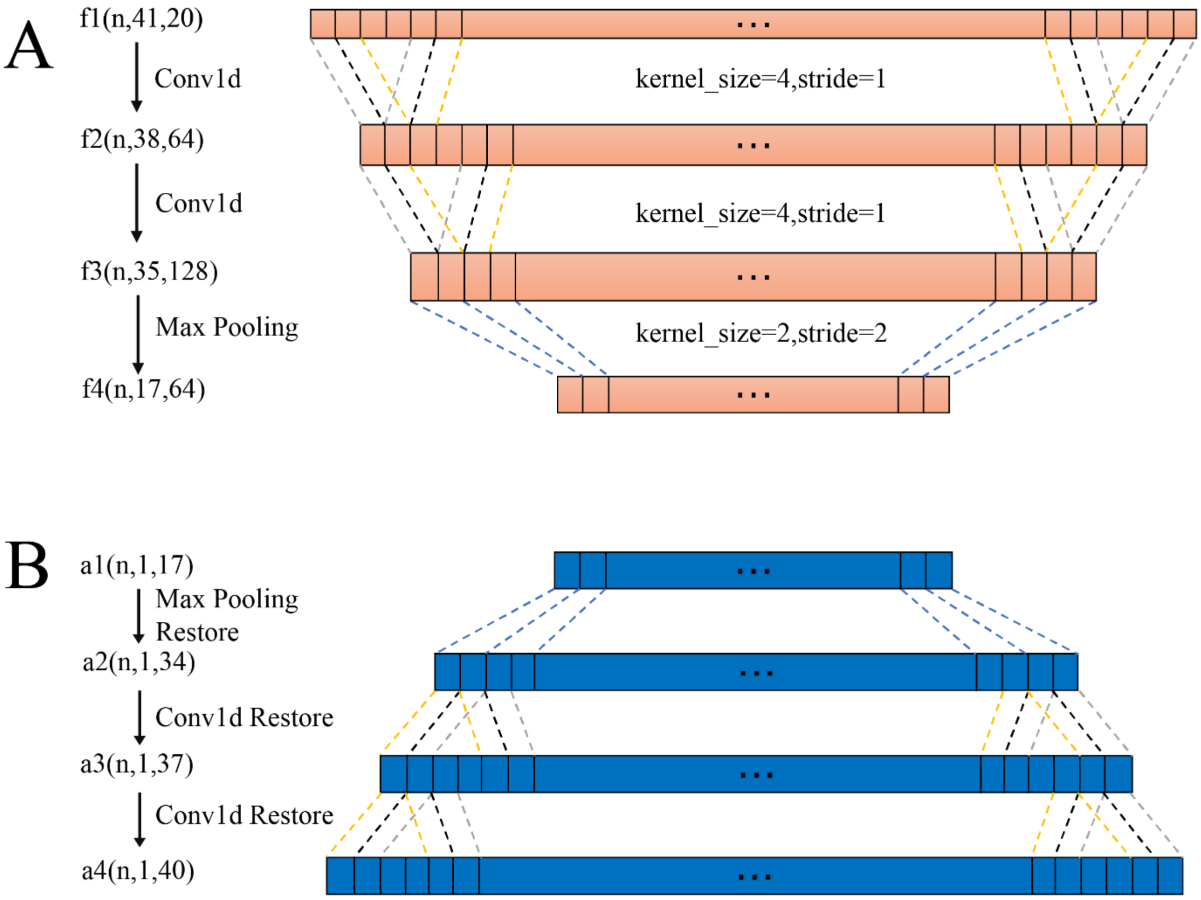

Figure S1 (A) shows the changes in the original feature matrix during the CNN module process, (B) shows the "stretching" process of the attention weight vector.
